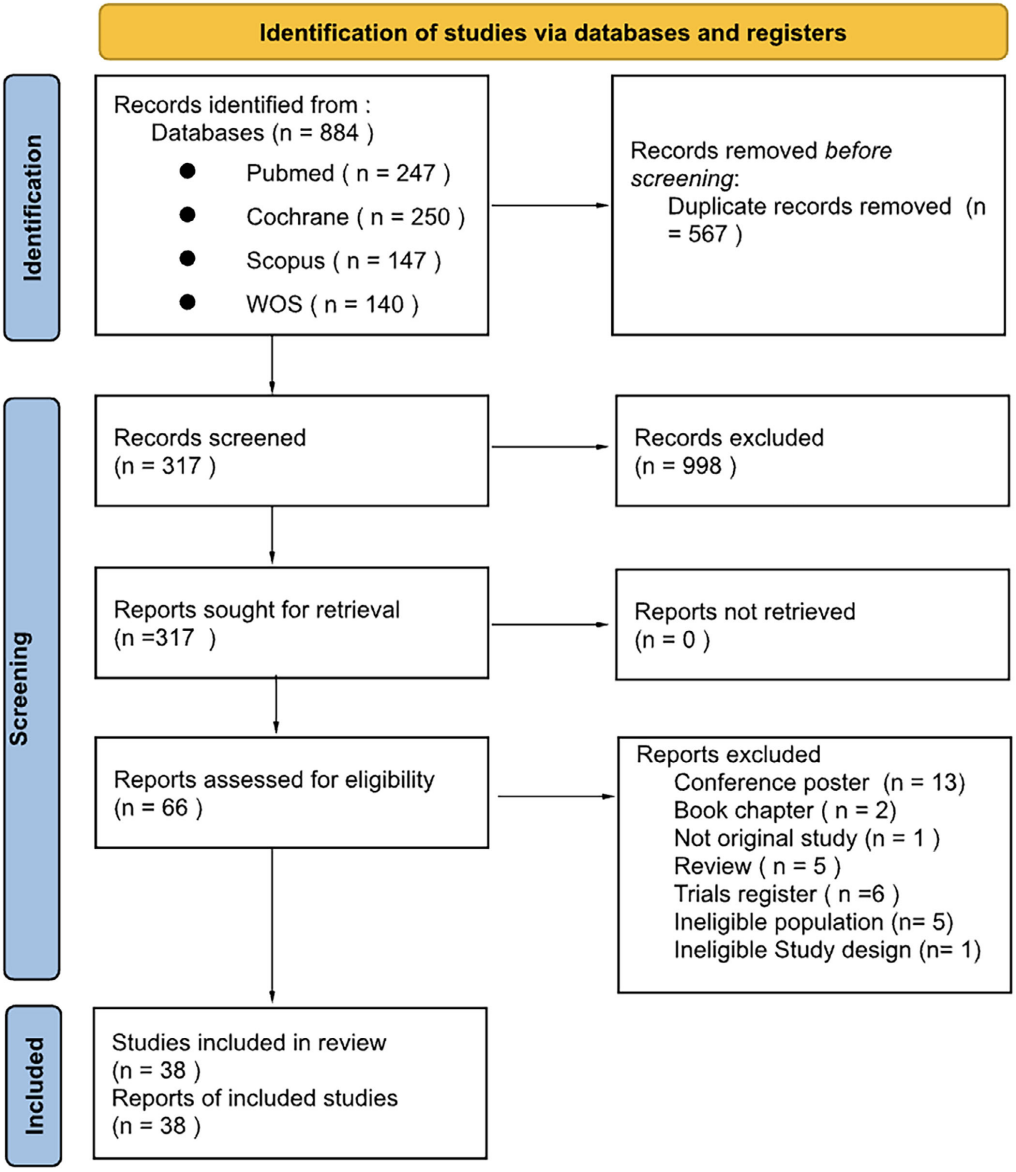# The effectiveness of dementia prevention programs: A systematic review and Meta analysis for 61,457 patients

**DOI:** 10.1002/alz.084229

**Published:** 2025-01-09

**Authors:** Ali Saad Al‐Shammary, AMEER FADHIL ABBAS AL WSSAWI, Hashim Talib Hashim, Fdel fadhel

**Affiliations:** ^1^ Baghdad medical city, Baghdad Iraq; ^2^ Al‐Qadisiyah university / College Of Medicine, بابل Iraq; ^3^ University of Warith Al‐Anbiyaa, College of Medicine, Baghdad Iraq; ^4^ Al‐Imam Al‐Sadiq Teaching general hospital, Hilla Iraq

## Abstract

**Background:**

Prevention programs for dementia are gaining increasing attention as the global population ages. These initiatives aim to reduce the risk of developing dementia by promoting brain health and implementing lifestyle changes. Through strategies such as regular physical exercise, a balanced diet, cognitive stimulation, and managing chronic health conditions, these programs strive to protect cognitive function and delay the onset of dementia. With the growing prevalence of this debilitating condition, investing in dementia prevention has become a crucial public health priority.

**Method:**

**884** records were screened based on our search strategy on main database (Pubmed/ MIDLINE, Scopus, WOS, Cochrane). After remove the duplicate and meticulous assess for eligibility **38** RCT included in our review.

**Result:**

38 RCTs and qualitative studies were included. **61,457** patients were included in all the studies. The ages were ranging from 60 years and above. We found that the most effective programs are ACTIVE diet and FINGER diet. ACTIVE program affecting mainly the reasoning and memory interventions primarily and secondarily the HRQoL, mobility and health services use. While FINGER program affecting global cognition and daily functioning in older people.

**Conclusion:**

**prevention programs for dementia play a pivotal role in promoting cognitive health and enhancing the quality of life for individuals as they age**. These programs encompass lifestyle modifications, cognitive training, and early detection efforts that empower individuals to take proactive steps in reducing their risk of dementia.